# Clinical impact of AI in radiology department management: a systematic review

**DOI:** 10.1007/s11547-024-01880-1

**Published:** 2024-09-07

**Authors:** Elvira Buijs, Elena Maggioni, Francesco Mazziotta, Federico Lega, Gianpaolo Carrafiello

**Affiliations:** 1 University of Milan, Via Festa del Perdono 7, 20122 Milan, Italy; 2 IRRCS Ospedale Policlinico Ca’Granda, Via Francesco Sforza 35, 20122 Milan, Italy

**Keywords:** Administration, Artificial intelligence, Clinical impact, Management, Organization, Radiology

## Abstract

**Purpose:**

Artificial intelligence (AI) has revolutionized medical diagnosis and treatment. Breakthroughs in diagnostic applications make headlines, but AI in department administration (admin AI) likely deserves more attention. With the present study we conducted a systematic review of the literature on clinical impacts of admin AI in radiology.

**Methods:**

Three electronic databases were searched for studies published in the last 5 years. Three independent reviewers evaluated the records using a tailored version of the Critical Appraisal Skills Program.

**Results:**

Of the 1486 records retrieved, only six met the inclusion criteria for further analysis, signaling the scarcity of evidence for research into admin AI.

**Conclusions:**

Despite the scarcity of studies, current evidence supports our hypothesis that admin AI holds promise for administrative application in radiology departments. Admin AI can directly benefit patient care and treatment outcomes by improving healthcare access and optimizing clinical processes. Furthermore, admin AI can be applied in error-prone administrative processes, allowing medical professionals to spend more time on direct clinical care. The scientific community should broaden its attention to include admin AI, as more real-world data are needed to quantify its benefits.

**Limitations:**

This exploratory study lacks extensive quantitative data backing administrative AI. Further studies are warranted to quantify the impacts.

## Introduction

Artificial Intelligence (AI), one in a series of disruptive technological innovations, is defined as the ability of a machine or software to perform cognitive tasks that simulate human intelligence. [1] Programmed to think and learn like people, AI uses extensive datasets to solve problems and perform tasks that typically require human intelligence. First applied in radiology in 1998 as computer-aided diagnosis software to detect microcalcifications in mammography, AI has gained much recent attention. [2] Current applications now include narrow task-specific intelligent systems used as auxiliary tools for radiologists, for example in identifying pulmonary nodules in image-based screening and categorizing them as benign or malignant. [3, 4]

Radiology has pioneered the potential of AI, given its track record in leading digital transformation in healthcare and meeting the demand for greater efficiency. The challenges in radiology derive from the volume, clinical utility and cost of rapid imaging services, which also drive AI development and applications.

Alongside the development of clinical applications of AI in radiology, there is increasing opportunity for non-clinical applications that have perhaps been overlooked. Non-clinical AI solutions, or admin AI as we define it here (where administration involves organization, operations and management practices) [5], are designed to optimize organizational processes within a radiology department. This can have a direct effect on clinical outcomes, such as greater access to healthcare and appropriate assignment of resources. These solutions are especially relevant for radiology, given the high volume of costly diagnostic tests and interventions.

So far, applications have been limited to narrow tasks in screening tests because of the lag between research and implementation due to lengthy procedures, especially in regulatory applications (e.g., U.S. FDA), clinical software integration, and reimbursement. Clinical AI systems have not enhanced productivity as might have been expected and their economic advantage has not been demonstrated. [6] Yet, given that administrative cost reduction is one among many ways to contain overall healthcare costs, admin AI systems, which do not require cumbersome procedures like clinical AI solutions, are a promising area for achieving gains in the organization of patient care in hospitals and their radiology departments.

## Focus and methods

Databases were chosen based on their relevant contents for medical and health management records (PubMed, Embase, Scopus). Main systematic review databases were consulted to prevent duplication (Cochrane, PROSPERO). Gray literature was not considered for the systematic review but used for discussions of its findings. To better understand the scope of admin AI in radiology, we (E.B., E.M., F.M.) conducted a systematic review of the literature and independently reviewed the records retrieved from chosen databases and randomly assigned for initial screening. The remaining records were evaluated against inclusion and exclusion criteria. At the end of each step, we shared our results and the selected records were independently read and evaluated. Discrepancies concerning the scoring of papers among the reviewer were discussed until consensus was reached.

The records included in the review at the end of the second step were evaluated according to a tailored version of the revised Critical Appraisal Skills Program (CASP), a standardized and validated checklist. [7] The Preferred Reporting Items for Systematic Reviews and Meta-Analyses (PRISMA) statement method [8] was used to transparently communicate the procedure for consulting the databases up to the final list of studies.

## Research objective

This systematic review of recent literature focused on AI applications for organizational purposes rather than strictly clinical AI such as image-based diagnosis support software. To do this, we investigated the applications being developed and applied, their quantitative results, and perceived benefits for a hospital organization and treatment outcomes. A further aim was to build a conceptual framework of the benefits of AI in administrative processes and to better understand the future of these innovative solutions for a radiology department, radiologists, and clinical decision makers.

## Research string

**Fig. 1:**
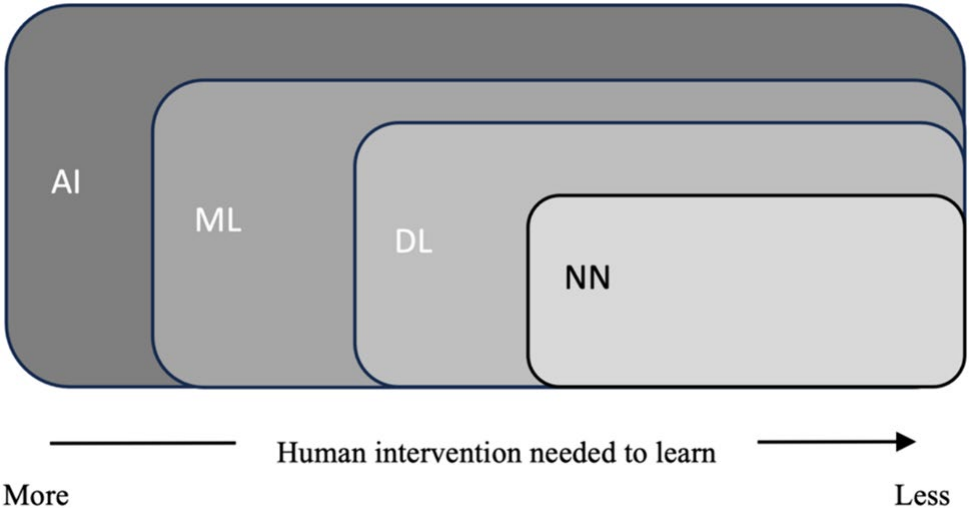
AI and its subfields

Considerable confusion surrounds the definition of AI. The terms AI, machine learning (ML), deep learning (DL), and neural networks (NN) are often used interchangeably but they are not synonymous; in fact, each is a component of the wider term (Fig. 1): ML is a subfield of AI, DL is a subfield of ML, and NN make up the backbone of DL algorithms. Moreover, NN mimics human brain processes by means of a set of algorithms in which DL refers to the depth of layers in a neural network. ML is more dependent than DL or NN on human intervention to learn. [1]

Because of this complexity, we paid close attention to the choice of MeSH terms to be included in the search string. The items (machine learning) and (neural network*) were included as subsets of the concept of (artificial intelligence); whereas, the terms ‘digitalization’ and ‘automation’ were excluded because they do not fall within the concept of AI. The definition and the scope of AI were set, and the search was limited to the scope of AI application to administration or organizational processes only. We decided to not include the term "management" in the search string because it is often used in the context of clinical cases and often inappropriately used by non-English-speaking literature. Finally, we excluded any locutions attributable to clinical outcomes: (diagnostic*), (clinical decision support), (disease detection), and (clinical management).

The databases were queried on 13 April 2023, with the search string composed of free terms, truncated word (*), and Boolean connectors (Table 1). Moreover, we narrowed the search field by defining within the string where each term should appear. Specifically, the search was limited to Title and Abstract or to Title alone. Managerial scope was searched using the restriction of “administration”, “management”, “planning” and “operation*” terms appearing in the title. The clinical approach was deleted both from the title and the abstract to further restrict the search.

**Table 1:** Research strings by database

Database	Research query
PubMed	((((((((((((((artificial intelligence[Title/Abstract]) OR (machine learning [Title/Abstract])) OR (neural network* [Title/Abstract])) AND (administration[Title]» OR (managementfTitle])) OR (planning[Title])) OR (operation* [Title])) NOT (digitalization[Title/Abstract])) NOT (automation[Title/Abstract])) NOT (diagnostic*[Title/Abstract])) NOT (clinical decision support[Title/Abstract])) NOT (disease detection[Title/Abstract])) NOT (clinical management [Title/Abstract])) NOT (disease management[Title/Abstract])) AND (radiology[Title/Abstract])
Embase	TITLE-ABS ( ( artificial AND intelligence) OR ( machine AND learning) OR (neural AND network*) AND ( radiology)) AND TITLE ( ( administration) OR ( management) OR ( planning) or (operation*))AND NOT TITLE-ABS ( ( digitalization) AND (automation) AND ( diagnostic*) AND ( clinical AND decision AND support) AND ( disease AND detection) AND ( clinical AND management) AND ( disease AND management))
Scopus	TITLE-ABS ( ( artificial AND intelligence) OR ( machine AND learning) OR (neural AND network*) AND ( radiology)) AND TITLE ( ( administration) OR ( management) OR ( planning) or (operation*))AND NOT TITLE-ABS ( ( digitalization) AND (automation) AND ( diagnostic*) AND ( clinical AND decision AND support) AND ( disease AND detection) AND ( clinical AND management) AND ( disease AND management))

In all three databases, filters applied to the research strings, including the publication date of the last five years, (2018–2023), English language, and human species

### Inclusion and exclusion criteria

The studies evaluated the application of AI in a radiology department with implications for departmental organization or administration (patients, payments, personnel, healthcare assets). The results of the studies were expressed in both quantitative and qualitative terms. A further inclusion criterion was a description of future trends for admin AI in radiology. Studies which only investigated or described clinical impact were analyzed to determine whether their results could also have consequences for research. There were no restrictions on the type of study or type of record. Studies that did not meet the inclusion criteria were excluded. Specific exclusion criteria were defined for studies which included only AI applications of a strictly clinical subtype (e.g., diagnostic support, image-based AI for diagnosis) or whose implications did not have organizational repercussions or which discussed a technological solution that did not meet criteria to be defined as AI. A distinction was made between clinical AI applications in which clinical diagnostic technology was used to optimize workflow, for example by eliminating negative results and prioritizing positive results. Studies solely describing clinical AI applications to aid diagnosis were excluded. All types of published studies were considered. Correspondence was specifically evaluated for scientific depth and breadth of the topic discussed before being deemed eligible for inclusion. Lastly, records not retrievable online were excluded.

**Fig. 2:**
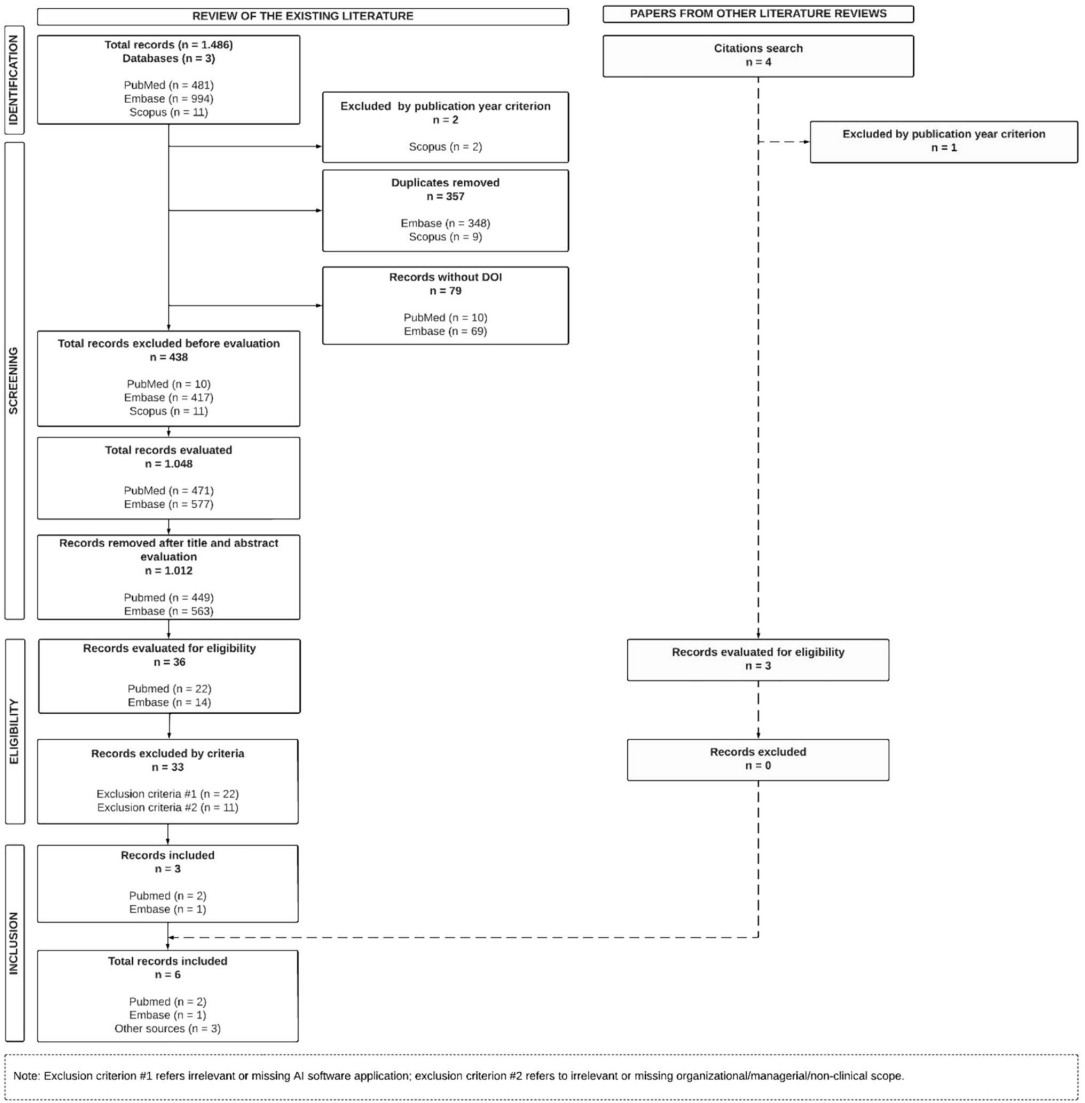
PRISMA flowchart

We independently screened the titles and abstracts of all records after removing the duplicates. We discussed definitive inclusion or exclusion, then independently reviewed the full text of the included studies. An overview of screening and inclusion in the study is presented in Fig. 2.

### Data extraction

Relevant data were extracted for: main author, year of publication, and type of study. Specific information regarding AI included a description of AI, medical field of application (i.e., emergency radiology, radiology, biomedicine, etc.), care pathway (i.e., triage, diagnostics, after care, scheduling, etc.), and technological aspects of the AI system (Table 3).

The methodological quality of the studies was evaluated using the revised CASP appraisal checklist [7]. As various types of scientific articles and studies were included, the evaluation options of the CASP checklist were modified to determine whether an element could be considered relevant to the type of study, adding outcome option D (Table 2).

**Table 2:** Elements reviewed using the CASP method

Item	Element	A/B/C/D score
1	Clarity of the research objective or of the research question	
2	Coherence in methodology	
3	Appropriateness in recruitment	
4	Type of data and collection	
5	Other literature analysis	
6	Analysis of contrasting data	
7	Sensitivity analysis of bias	
8	Coherence of results with the research question	
9	Consideration of ethical issues	
10	Presented model replicability	

The four outcomes of evaluation were:

A. Considering the type of study examined, the element is properly or extensively covered.
B. Considering the type of study examined, the element is partially covered.
C. Considering the type of study examined, the element could have been covered or, at least, considered but it is not.
D. Considering the type of study examined, whether the element is relevant to the study to be covered or considered.

Two of us independently evaluated the studies and then ranked them based on the percentage of criteria fulfilled. Depending on the type of study, we did not consider the elements classified D (whether the element is relevant to the study to be covered or considered) to affect this percentage. A record was judged having high quality if the percentage of elements ranked with an A (the element is properly or extensively covered) was more than 60% and the percentage of C (element could have been covered or, at least, considered but it is not) was less than 20%. Records were considered qualitatively low if the percentage of the A elements was less than 20% and the percentage of the C elements was more than 40%. All other combinations were ranked as moderate. While the rank did not exclude any records, those with low scores were treated differently in the discussion.

## Results

### Search results

Screening the three databases (PubMed, Embase, Scopus) yielded a total of 1486 records, two of which were excluded because they were published before 2018; 357 duplicates were removed; 79 records lacked a DOI, making them unretrievable for full review. The remaining 1048 records were screened by title and abstract; 33 of the 36 selected for further analysis were excluded. The remaining 3 records met the inclusion criteria. Furthermore, other records gleaned from the references were identified as possibly relevant and were reviewed for eligibility via a search of citations. Three records were included in the systematic review (Fig. 2). The data were extracted, and two of us independently assessed the records for quality.

### Quality assessment

**Fig. 3:**
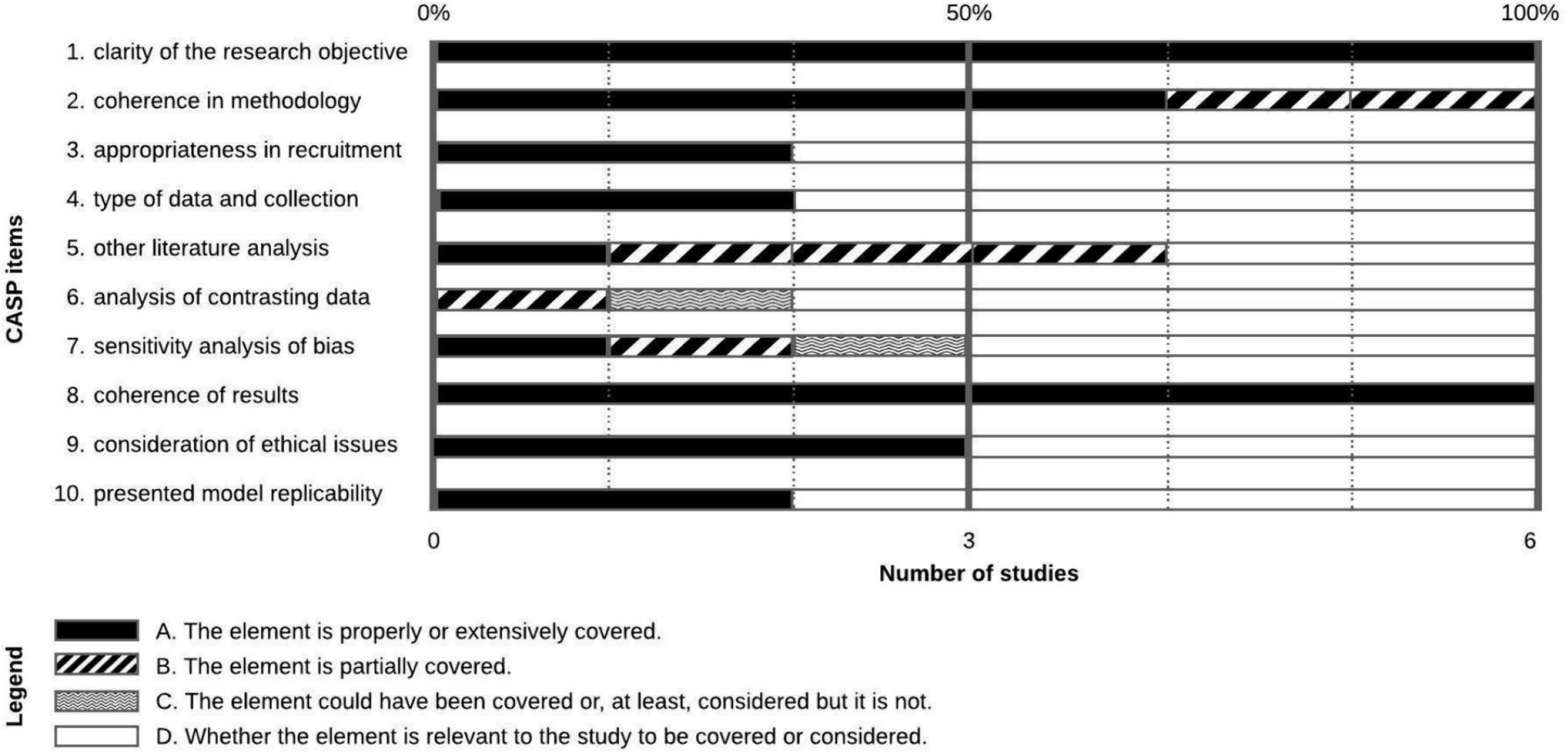
Quality Assessment

Quality assessment was performed using a set of questions from the CASP method. The score was calculated as the percentage of criteria fulfilled, and the records were ranked accordingly (Fig. 3).

In their correspondence, Berlyand et al. [9] reported evidence from a large reference dataset of studies to identify which AI interventions and subsequent operations can benefit emergency department workflow. A possible bias of the study was that author affiliation and geographical location of the evidence were the same. In their original research articles Chong et al. [10] and Verburg et al. [11] reported evidence from large patient datasets, with clear and concise methodologies. To conclude, the literature reviews [12–14] focused primarily on the future role of AI in the governance of radiology departments and the implications for healthcare professionals. Moreover, the studies analyzed how AI optimized workflow and clinical outcomes, with strong implications for management and organizational processes.

### Findings

Table 3 presents an overview of the studies and details on AI applications. In detail, the studies were published between 2018 and 2022 with no predominance of any year and included literature reviews [12–14], original research articles, [10, 11] and correspondence. [9] All the studies discussed AI applied to radiology, both emergency radiology [9, 14] and elective radiology, [10–13] consistent with the inclusion criteria. The study on magnetic resonance imaging (MRI) as a diagnostic technique [10, 11] appeared relevant, as both original research articles investigated this. The stage in the care path in which AI is most often applied and investigated is appointment scheduling [10, 12, 13] and triaging. [9, 11]. Furthermore, the main types of AI were ML [9, 10, 12, 13] and DL. [11, 12]

**Table 3:** Study Characteristics

Main author	Year of publication	Type	Description of AI application	Medical field	Care pathway phase	AI technology
Berlyand [25]	2018	Correspondence	- Triage and outcome prediction to match resources to patients’ needs	Emergency medicine, Emergency radiology	Triage, diagnostics, after care	Machine learning, Natural Language Processing (NLP)
			- Exclusion of life-threatening pathologies on non-contrast head CTs			
			- Fracture recognition to facilitate disposition and accurate resource allocation			
Syed [22]	2018	Review	- Patient scheduling, workflow optimization through abnormality detection and prioritization	Radiology	Scheduling	Machine learning, image detection, NLP, deep learning, segmentation
			- Flagging non diagnostic exams to repeat			
			- Optimization of prior studies in PACS			
			- Billing and coding			
Ho [21]	2019	Review	- Precision scheduling	Healthcare, Biomedicine, Radiology	Scheduling, diagnostics	Machine learning, others not specified
			- Identification of patients who are likely to miss appointments			
			- Customized examination protocols			
Chong [24]	2020	Original research	- Outpatient appointment no-shows on 32,957 appointments, predictive model to deploy telephone reminders	Radiology, MRI	Scheduling	Machine learning predictive analytics
Scheinfeld [20]	2021	Review	- Streamlining workflow through case prioritization	Radiology, Emergency radiology	Screening, diagnostics	Segmentation, image detection
Verburg [23]	2022	Original research	- Optimizing workflow and reducing workload through identification of negative breast MRI in 4581 MRI studies while identifying malignant disease	Radiology, MRI, Senology	Triage	Deep learning

PACS denotes picture archiving and communication system; MRI magnetic resonance imaging

Current AI admin solutions are generally employed to reduce fluctuations in healthcare demand, which are more prevalent in non-elective settings such as the emergency room. As a consequence, the focus was on triage and scheduling in admin AI.

#### Triage and patient stratification

One benefit of AI for radiology departments is that it provides for faster interpretation of clinical data for patient classification during triage and clinical risk assessment during discharge [11, 13, 14]. Accurate and rapid triage based on highly sensitive algorithms can help to distinguish critically ill from stable patients [9] and predict response to therapy through “big data” data techniques, consisting of DL and other ML strategies. [12]

In addition, natural language processing (NLP) and ML models have been developed that can identify illnesses (e.g., sepsis, acute appendicitis, influenza) and perform computer-aided diagnosis with extraordinarily high accuracy using data typically available within hours of an emergency room visit. [9, 13] This type of analysis allows for better allocation and use of resources, patient management, and operations especially in urgent care. Such improvements allow for better matching of resources with patient needs, thus increasing efficiency in time and cost and improving patient outcomes. [9, 11, 12] Improvement in patient outcomes results from averting delays and potential worsening of illness, appropriately assigned resources, and data-backed care. Given the aging population in many developed countries and the rising costs of healthcare delivery, there is an imperative need to keep healthcare both accessible and affordable [10].

In addition, the workflow of specialists and the management of emergencies during elective appointment hours can benefit greatly from triage, faster risk stratification and identification. AI models can accurately predict wait times or appointment delays for computed tomography (CT), MRI, radiography, and ultrasound. The ability to communicate accurate waiting times to patients can improve patient satisfaction. [12]

#### Scheduling and workflow

Hospital outpatient appointment no-shows are a common problem and a costly burden to healthcare systems worldwide, as they contribute to inefficiencies and health access delays [10, 13]. Recent studies in appointment scheduling indicate that overtime, idle time, and patient waiting time can be substantially reduced by combining ML with a framework called predictive overbooking. [10, 15] In fact, ML techniques trained using high-dimensional datasets to produce complex high-performance predictive models can manage the multifaceted problem of appointment no-shows. [10] Moreover, studies agree that AI can benefit through worklist prioritization, double-checking of errors (i.e., misses), and predicting patient volume in the emergency department. [14, 16]

Furthermore, AI algorithms can be useful in providing guidance for selecting which imaging exam may be most appropriate (e.g., MRI rather than ultrasound based on patient characteristics). [12] Greater appropriateness of care and simultaneous reduction in idle time is likely to improve patient and staff satisfaction.

#### Claim and reimbursement processing

Many variables can figure in a denied claim; AI can aid in coding and billing procedures. AI applications can positively impact on staff satisfaction in such tedious and repetitive tasks as medical coding. [12] For radiologists, less time spent on administrative tasks can free up time for diagnostics and patient care, both beneficial to clinical outcomes.

## Discussion

**Fig. 4:**
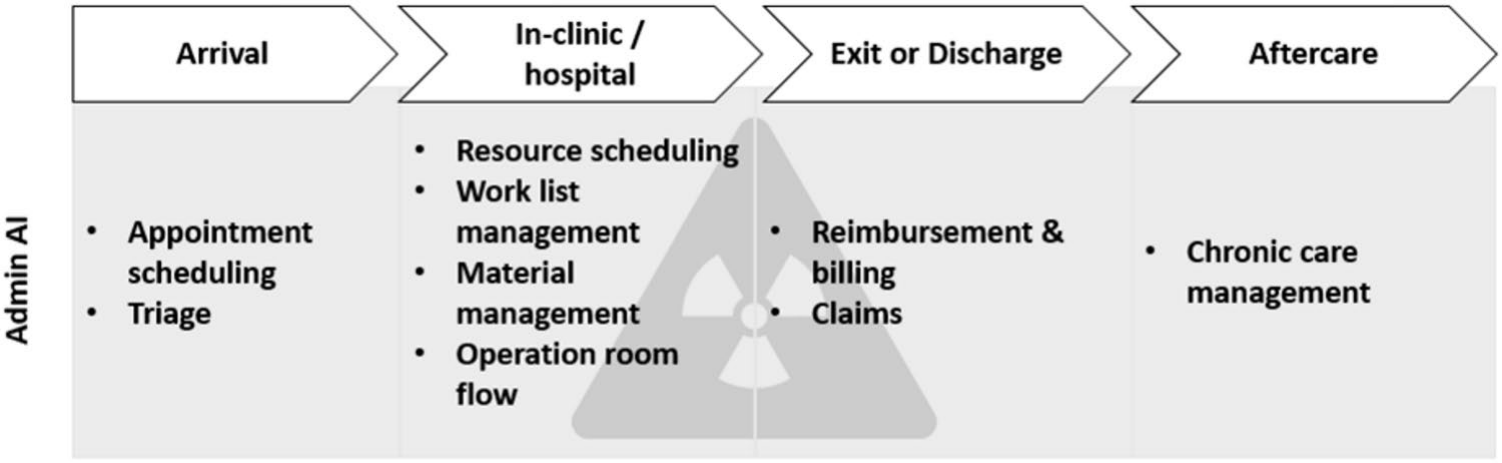
Admin AI applications in (interventional) radiology along the patient journey

Despite the limited data on admin AI in radiology, this systematic review found encouraging evidence for the future. Indeed, the current literature highlights the potential of admin AI in various settings. Building on this, we developed a framework summarizing the main applications of admin AI categorized by elements of the patient pathway (Fig. 4).

Before we discuss strategic and operational implications for hospital managers and decision makers, three issues in the implementation of AI need to be addressed: ethical implications, costs, and barriers. [17, 18]

### Ethical implications and overall satisfaction

Appointment scheduling and management of agendas are among the main factors that impact efficiency in radiology departments. AI in complex predictive models can identify appropriate exams in advance [10, 12–14]. The reduction in physician idle time and patient waiting times (and waiting lists) results in greater satisfaction for staff and patients alike [19]. Furthermore, streamlining patient pathways and emergency radiological diagnostic procedures may lead to better health outcomes [14, 16].

Unless well managed, however, AI can have serious ethical implications. Though AI is ethically neutral, human programmers can unintentionally introduce cognitive and algorithmic biases that influence its effectiveness. AI reflects the quality of the data it is trained on. For example, minorities who are not well-represented in the original data set could lead to a racial bias in the software. When the objective is to minimize scheduling costs, unintended racial disparities may ensue: for example black patients had to wait about 30% longer than nonblack patients. [15] This bias becomes particularly evident in multiracial contexts. Hence, when introducing AI into their organization, managers need to ask themselves critical questions and adopt race-aware objectives. Additionally, healthcare organizations should test algorithms in real-life settings and adopt a human-in-the-loop, where humans provide consistent feedback from which an AI system can learn. [13] More importantly, the demographics of the population used for AI training need to be aligned with the target population. [15, 20, 21]

Healthcare organizations can also use anonymized patient data, but the ever-increasing complexity of AI systems requires greater amounts of data to learn from. This means a risk for anonymization and that private companies (mis)use the data required by the machine. [20] Legislative effort is therefore also needed by individual countries to enact privacy legislation and promote the efficient use of new technologies.

### Costliness of applying admin AI

The adoption of AI by healthcare organizations entails considerable economic investment. Correctly evaluating AI economically is especially difficult because it is currently impossible to capture the dimensions of complexity and clinical applicability to support appropriate decision making. Therefore adequate AI evaluation models need to be defined. [22] In addition to the direct costs attributable to the technology, healthcare managers must also consider indirect costs and time for staff training [23]. Personnel directly involved in the use of AI and personnel who will use the administrative and management reports generated by the AI must be adequately trained and updated.

Furthermore, healthcare organizations wanting to implement AI in their radiology department need to be aware of the drawbacks of being an early adopter [24, 25]. Besides the high costs of new technology and the ancillary costs for personnel training, early adoption involves costs related to the compatibility between the new and the old technology present in the organization. This phenomenon is described for new technologies, where first-generation technologies have not yet generated their full breadth of benefits, with the risk that the technology soon becomes obsolete. Second-generation technologies might overcome certain issues and may incentivize investments early rather later on [26].

Nonetheless, being an early adopter also has its advantages, e.g., high visibility for a healthcare organization. Furthermore, innovators can be a relevant resource for companies developing AI by allowing them to refine and optimize the product, providing a competitive advantage. [24]

### Barriers to introduction

As with all breakthrough innovations, AI tools must overcome barriers to implementation, such as the cost of adoption and training, hesitancy of clinicians to accept job changes, increased job standardization and consultative professional leadership in organizational governance. [9, 13] Moreover, ML models need periodical re-evaluation with updated data on institutional workflow practices, patient demographics, and equipment. [10]AI is an investment that needs resources to generate validated output. Furthermore, some models are more likely to be implemented in the short run than others due to data availability. [9]

Nevertheless, ethical implications can pose a barrier to the full potential of AI in healthcare. Effective governance is key to stepping up to the many challenges of AI and to counter disincentives and slowdowns during its adoption. [13]

To conclude, technological heterogeneity and incompatibility among hospital systems may generate hesitation among medical providers relying on AI. [9] Current ML solutions have narrow application to specific cases and are often tailored to the needs of the facility. [10]

## Implications for clinical managers and decision makers

**Fig. 5:**
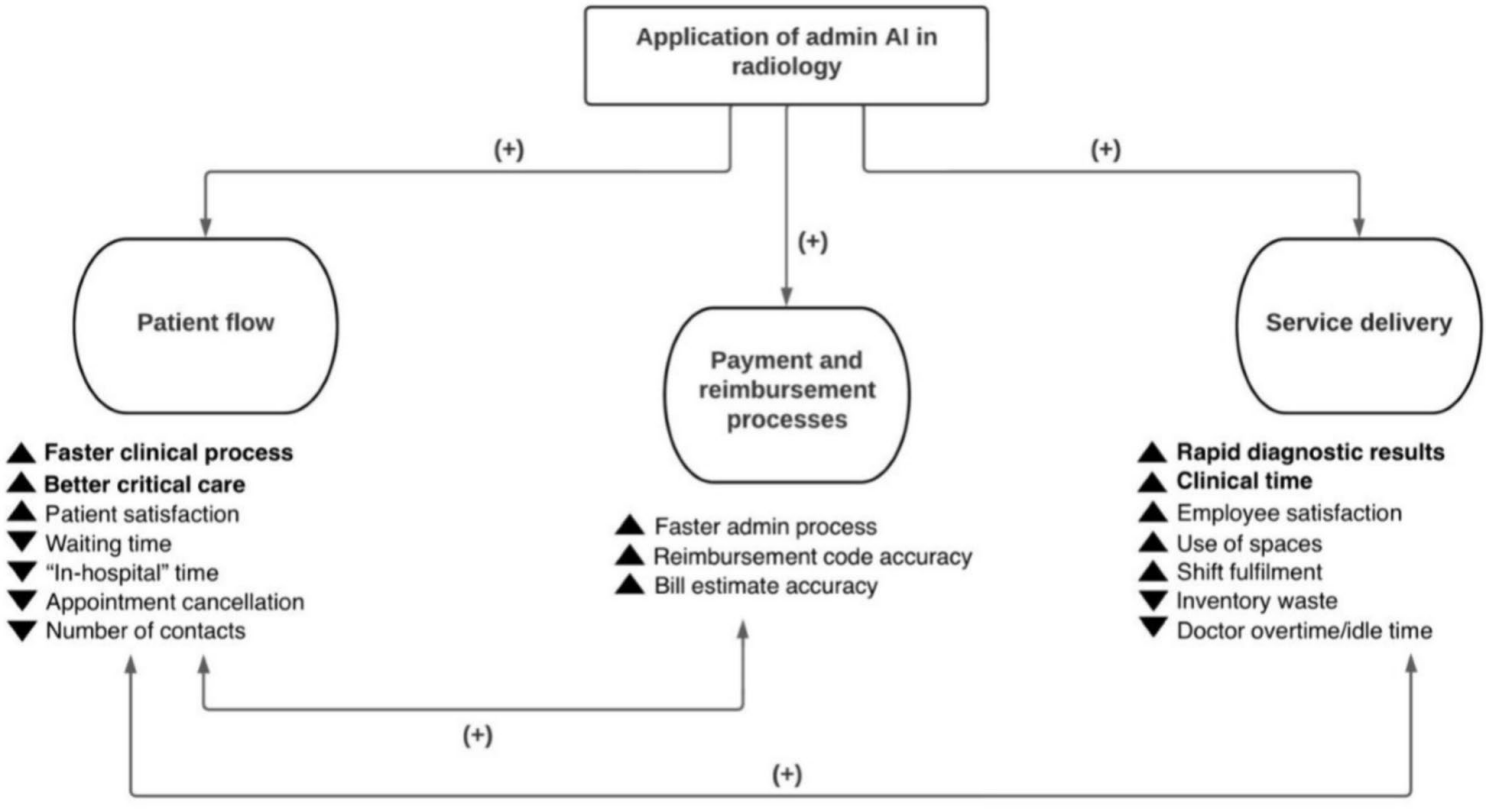
Concept map of implications. *Note*: Main clinical impacts are shown in bold

Implementing AI in a hospital radiology department can impact on performance and operations. Evidence from this review suggests a conceptual framework that systematizes potential AI implications (positive or negative) for the management of a radiology department in three dimensions: patient flow, payment and reimbursement processes, and service delivery (Fig. 5).

### Patient flow

The demand for imaging services has outpaced the resources needed to expand production capacity. [27, 28] This leads to bottlenecks in the flow of elective and emergency patients and in longer waiting lists. [29] The wait generates multiple non-indifferent externalities for inpatient or outpatient admission and care, both from a clinical and an administrative perspective. [28, 30] Prolonged waiting and delays can exacerbate a patient’s clinical condition. Given the greater complexity of care, this implies that more resources be dedicated to the clinical case by the healthcare system (physicians, drugs, therapies, time) and the patients themselves (time and resources, drugs and therapies, personal economic losses). This, in turn, reduces patient satisfaction, with a shift of resources from the public to the private sector. Well-implemented AI, however, may predict clinical complications, estimate fluctuation in demand, and identify in advance which patients will require more resources, with a direct effect on quality of care and care outcomes (appropriateness) [31]. From a managerial perspective, these clinical impacts translate into shorter in-hospital stay and prevent against appointment prolongation from disrupting the overall agenda. [32] Such variables could improve patient and caregiver satisfaction. [27, 33]

Furthermore, the predictive capacity of AI allows for improved appointment scheduling by considering the probability that a patient cancels the visit or is a no-show. [27, 33] This improves the management of healthcare staff agendas and the use of limited resources, such as expensive machines, technology, and ultimately space. In daily clinical practice, this could translate into a reduction in idle time of healthcare personnel and in the need to schedule overtime due to ill-planned agendas and spaces. In quantitative and economic terms, this can increase the efficiency of resources spent for the delivery of services, with a reduction in long-term management costs. [27]

The improvement in staff time management within an innovative work context can increase personnel satisfaction and sense of empowerment. Moreover, studies that found a link between healthcare worker job satisfaction and overall patient satisfaction and a more patient-oriented approach to everyday tasks [34–36], in which a virtuous circle of satisfaction is generated among stakeholders.

### Payment and reimbursement

One of the elements that most affect the economic sustainability of the system is the payment and reimbursement process. Artificial intelligence, by virtue of its predictive capacity of complications and patient no-shows, allows for better economic reporting and accounting [10, 32].

### Service delivery

A further aspect of the economic sustainability of healthcare organizations is the reduction of waste. Just as optimization of appointment scheduling involves predictive methods to reduce fluctuation in healthcare demand, so, too, inventory and inventory waste can be tackled in a similar manner. New storage tools based on AI and ML can help organizations with software that tracks expiring materials and aligns goods procurement with real needs through big data analysis. [10] This improved management of resources can also have direct clinical outcomes as materials used receive more careful monitoring.

## Conclusion

Implementation of admin AI in the radiology department has the potential to drastically change and improve workflow and clinical outcomes as well as other benefits for organizational processes. Radiologists should be excited about these new technologies on the cusp of change in professional activities, with the goal to improve diagnostic and therapeutic care for their patients. Further, the applications of AI discussed so far will have an impact not only on the radiology department but on the entire healthcare organization as well. Admin AI applications developed for the radiology department could be easily translated to other departments of a healthcare organization (e.g., billing and reimbursement, scheduling, etc.). Our findings and the framework we developed can be helpful in this respect.

Finally, the growth of the AI market in the healthcare sector in the last decade has been accompanied by an increase in the publication of scientific studies; however, few have focused on AI solutions for hospital administration and management. Despite the potential of admin AI highlighted in the current literature, we found little evidence of improvement in terms of quantitative outcomes. More research is needed to quantify the potential benefits of admin AI via longitudinal studies and randomized controlled trials to obtain reliable evidence for its implementation.

### Limitations

The systematic review, exploratory in its nature, lacks extensive quantitative data supporting the application administrative AI. As these technological developments are relatively new, further studies are warranted to quantify the impacts in their respective contexts.
